# Code of Ethics

**Published:** 1878-02

**Authors:** W. J. Chenoweth

**Affiliations:** Decatur, Ill.


### Code of Ethics.

By W. J. Chenoweth, M. D. Decatur, Ill.

The Code of Ethics is a standard of morals as binding on members of medical societies having representation in the American Medical Association, as is the creed of a religious society on its members. True, it does not claim to be inspired, but assent to its principles implies strict observance of its requirements, without the right of private interpretation. We cannot, therefore, join a society governed by the Code, and receive the benefits conferred by membership, and at the same time reap the advantages of an independent construction of its provisions to favor our inclinations or our interest. In case of differing in opinion with the majority, there are two modes of procedure, either of which is compatible with honor; one is to sever our connection with the society, the other to yield to the interpretation of its members and be governed by their views. We cannot afford to lose the benefits accruing from association by withdrawing; but as membership does not necessitate silence, we may seek to improve or annul any provisions which we deem obnoxious. The right of free speech is guaranteed to us in this, just as it is in every other society, and while we carry out in good faith the construction of the majority, we are not enjoined from expressing an individual opinion.

The design of its enactment being for the cultivation of the social qualities, and for the suppression of quackery, is hardly subject to criticism; but the degree of success which has attended it, and the intrinsic merits of its requirements, are ligitimate subjects of inquiry, demanding our serious consideration. Certain provisions requiring revision were noticed by J. Marion Simms in his inaugural address, May, 1876. Two others are sources of much difficulty—consulting with irregular practitioners and advertising. While the first is an expression of narrow policy and of short-sightedness in painful contrast to the liberal spirit of the age, we have not time in this report to notice it as we ought, and shall therefore pass it by and shall notice advertising alone. The Code, Art. 1st paragraph 3d, on ' The duties of physicians to each other, and to the profession at large" says "it is derogatory to the dignity of the profession to resort to public advertisements, or private cards, or handbills inviting the attention of individuals affected with particular diseases, publicly offering advice and medicine to the poor gratis, or promising radical cures; or to publish cases and operations in the daily prints, or suffer such publications to be made; to invite laymen to be present at operations, to boast of cures and remedies, to adduce certificates of skill and success, or to perform any other similar acts. These are the ordinary practices of empirics, and are highly reprehensible in a regular physician.

We will enquire first as to the success attained in the suppression of the various kinds of advertising noticed, and secondly as to the merits of the prohibition, and will endeavor to do this in the order in which they occur in the article.

The first clause aims to prevent specialists from resorting to public advertisements, and the American Medical Association has decreed that they shall not advertise in medical journals, so that advertisement of a specialty in a medical journal is just as much an infringement as the same would be in a newspaper. How then do specialists advertise, if at all? A common mode is to establish a private hospital, place it under the care of a religious society, and advertise themselves as consulting physicians. This is done in nearly all of our large cities, and while there is a deal of seeming modesty in it, it is so effectual that he is blind who cannot see it. Another mode quite as effectual is by reprinting essays which have been contributed to medical journals, and sending them to physicians and druggists all over the country. A third mode is to print the announcements of medical colleges, with the names and professorial chairs of the faculty in the prominent daily newspapers. But whatever the mode adopted there is no question of the fact that they do advertise, and so effectually as to be known outside of the profession almost as well as in the profession.

But the construction placed on the paragraph in reference to advertising, does not prohibit authors or publishers from scattering vaunting advertisements broadcast; nor does it forbid publishers or editors of medical journals from issuing prospectuses in which they claim that their issues are made up of original contributions from the ablest medical writers; that they will make "reports of rare and interesting cases;" that "the journal will rank with the best periodicals in the country;" nor does it prevent the professors in colleges from sending out advertisements claiming superior advantages for clinical instructions, abundance of material for dissection, and if a new professor is added or supersedes another, a careful epitome of his claims are duly chronicled. Why this kind of advertisement is allowed and a modest statement of preparation and ability as a practitioner forbidden, is beyond my comprehension. If advertising is honorable in a teacher it is difficult to convince an ordinary mind that it is dishonorable in the person taught—if honorable in a writer, dishonorable in a reader. The reason assigned in the paragraph of the Code under discussion is that it is derogatory to the dignity of the profession—an assumption that it is degrading to do as a doctor that which can be done with honor as a teacher, writer or lecturer. The only other reason assigned is that "it is the ordinary practice of empirics." Since we have shown that it is also the ordinary practice of the best known men in the profession, it is difficult to feel the force of the argument, and even though we should be mistaken, and physicians in high places do not advertise, the fact that empirics do, is not conclusive as to the propriety, since they eat, drink, sleep, wear clothing, laugh, talk, and do other things in common with the rest of mankind, and so far as any expression by the profession is known, it is not criminal in us to do likewise. Percival, who got up the Code, was an Englishman, and did not wish to be considered as belonging to the vulgar class who earn bread by the sweat of the brow, but rather to the aristocracy, and, as was natural, aped the manner and expressions of the class. He, and men like him, think it "derogatory to their dignity" to be seen at the public market with baskets on their arms, "or to perform any other similar acts," as "these are the ordinary practices" of the common people.

We will now ask attention to the several prohibitions in the order of their occurrence in the paragraph, and first in reference to specialists. There are but few men who are such universal geniuses as to be equally skilled in all branches of medicine. Every physician will have certain branches to which he pays more attention than to others, and in so far as he does, may be said to be a specialist, even though a general practitioner. Valentine Mott was known as a surgeon, and if the term specialist would apply to any one it would to him, and yet his income was greater from general practice than from surgery. Prof. Hodgen, of St. Louis is a general practitioner, and yet he is called as consulting and operating surgeon all over the West, and to this extent is recognised a specialist in surgery.

The second clause forbidding us to "publicly offer advice and medicine to the poor gratis" is probably in compliance with the scripture injunction not to let our right hands know what our left hands do, but while no class in the community do so many unchronicled acts of charity, it seems strange that they alone of all charitable men should be forbidden to make public announcements of their willingness to treat the poor gratis. There are two classes of men who ought to treat the poor gratis—those just beginning to practice and who will reap benefits for themselves while they confer blessings on the poor, and those who, by inheritance or otherwise, do not need the fees so vital to most of their brethren, many of whom have large experience and ripe knowledge. It seems to me that to a rich physician the great pleasure of life would be to publish his willingness to treat the needy "without money and without price." Not even regular physicians declaim against Howard, whose life and money were given to paupers and criminals. The pleasantest recollection we have of Geo. McClellan is popularity and love earned by attention to the poor.

The enactment against promising radical cures is surperfluous, while it is constantly evaded by inference and implication, leaving abundant room for explanation in case of failure; policy prevents even quacks from openly resorting to it as a means of securing practice, since intelligent physicians are almost a unit in declaring that no man can foretell the result of any given case—and to legislate for ignorant knaves will result very much like the laws which were written in small characters and hung so high that they could not be read.

In reference to publishing cases in the daily prints: Since the demands of the press require the employment of so many reporters it is not possible for a physician doing any considerable practice to keep his name out of the newspapers, as instanced before. To any practitioner living in a small city of from ten to fifteen thousand inhabitants, self-defence demands that his cases be reported in the papers so that people outside the city may know that the quacks around the corner who publishes, with embellishments and improvements, all of his cases, is not the only person in the city who has cases to treat. The local reporters will get hold of the names of parties injured, and if in spite of the utmost efforts of the conscientious physician, who is willing to sacrifice reputation and money in obedience to the requirements of the Code, his name is, occasionally, noticed, as the attending surgeon, it is evidence "strong as proof of Holy writ" that he suffers such publication to be made; and every case reported without his name is as apt to be credited to the quack as to himself.

In reference to inviting laymen to be present at operations, nothing against it can be urged that will not, by a parity of reasoning exclude physicians from the manipulations of scientists and other skilled workers.

In regard to boasting of cures and remedies, the offense is one against manners, rather than morals and is equally applicable to gentlemen in every condition of life, and in every occupation. Boasting means simply an expression of the estimate placed by us on our ability or achievements, which proves to be greater than that awarded to us by our fellows, or than that merited by our acts. The very essence of quacking is claiming ability to do, without capacity to execute, while a just claim, however extravagant in appearance, does not detract from dignity or merit.

The clause condemning the use of certificates of skill and success having been constantly evaded by the reports of cases with the pledge, expressed or understood, of the author as to their correctness, cannot be enforced except literally, and if it should be, neither books nor journals could furnish us the knowledge we so much crave. This clause, like the last mentioned, is an offense against manners; but as contemplated as certificates are, an occasional offense of this kind might be permitted with good result as an appendage to a few medical or surgical reports.

In England the profession is crowded with one physician to one thousand people—in the United States the porportion is one to five hundred, and gradually increasing. Either physicians must be protected by law, or they must be permitted to fight their own battles; and since the law cannot take us under its sheltering wings, to the prejudice of other citizens, whether they are Homœpaths, Eclectics, Hydropaths or Thompsonians, I see no other plan than to do away with the restraints which bind us while our enemies steal our bread, but let the fight be even—then there will be a survival of the fittest.

